# Persistent circulation of Rift Valley fever virus lineage C in Rwanda, 2022–2025

**DOI:** 10.1016/j.onehlt.2026.101529

**Published:** 2026-08-01

**Authors:** Jean Claude Udahemuka, Hayley Cassidy, Leonard Schuele, Evodie Uwibambe, Methode Gasana Ngabo, Leandre Murhula Masirika, Reuben Sindayiheba, Saria Otani, Misbah Gashegu, Jean-Claude Twizere, Frank Aarestrup, Fabrice Ndayisenga, Bas B. Oude Munnink, Marion P.G. Koopmans, Pacifique Ndishimye

**Affiliations:** a Department of Veterinary Medicine, University of Rwanda, Nyagatare, Rwanda; b Stansile Research Organization, Kigali, Rwanda; c Department of Viroscience, Pandemic and Disaster Preparedness Centre, Erasmus University Medical Center, Rotterdam, the Netherlands; d Department of Animal Resource Research and Technology Transfer, Rwanda Agriculture and Animal Resources Development Board (RAB), Huye, P.O. Box 5016, Rwanda; e Congo Outbreaks, Research for Development (CORD), South-Kivu, Bukavu, Democratic Republic of the Congo; f Centre de Recherche en Sciences Naturelles de Lwiro, South-Kivu, Bukavu, Democratic Republic of the Congo; g National Reference Laboratory, Rwanda Biomedical Centre, Kigali, Rwanda; h Research Group for Genomic Epidemiology, National Food Institute, Technical University of Denmark, Lyngby, Denmark; i Université de Liège, Liege, Belgium; j Epidemic Response Laboratory, African Institute for Mathematical Sciences (AIMS), Kigali, Rwanda

**Keywords:** Rift Valley fever virus, Whole-genome sequencing, Metagenomics, Nanopore sequencing, Public health surveillance, Genomics

## Abstract

Rwanda has experienced recurrent Rift Valley fever virus outbreaks in the last decade. In this study, we investigated whether these outbreaks resulted from repeated introductions or sustained local circulation. We generated RVFV whole-genome sequences from livestock samples collected between 2022 and 2025 using Nanopore sequencing. Genomic analyses indicated the outbreaks resulted from sustained local circulation of lineage C rather than repeated introductions, suggesting ongoing transmission likely driven by sporadic spillover. This study underscores the importance of continuous genomic One Health surveillance in endemic settings.

## 1 Introduction

Rift Valley fever virus (RVFV) is a significant threat to human and animal health across East and Sub-Saharan Africa with increasing frequency and scale of outbreaks [1]. This is underscored by the 2025 RVFV outbreak in Senegal and Mauritania [2], affecting both humans and animals, with ongoing transmission reported as of January 2026 [3], [4]. Transmission typically occurs through *Aedes*, and to a lesser degree *Culex* mosquito bites or contact with contaminated blood, primarily affecting cattle, sheep, and goats [5]. This can result in fever, hemorrhaging and “abortion storms” leading to significant economic losses [6]. Humans can get infected through mosquito exposure, as well as handling of animal tissues and consumption of unpasteurized milk. Climate variability, land-use change and livestock movement may increasingly influence RVFV transmission across the human–animal–environment interface, underscoring the importance of a One Health approach to surveillance and control [5].

RVFV was first detected in Rwanda in 2012 and has since caused small annual outbreaks, culminating in larger outbreaks in 2022 and 2024 [7], [8], with further case numbers in 2025, affecting different regions within Rwanda*.* It is unknown whether the different outbreaks are linked to a single introduction or reflect repeated introductions or both. Here, we present the genomic characterization of RVFV from livestock with initial genome detection through metagenomic analysis from samples collected during the 2024 outbreak, and subsequent expansion of the study to include a wider representation of samples collected between 2022 and 2025 using real-time field-based Nanopore sequencing.

## 2 Materials and methods

Blood samples were collected from suspected livestock cases (cattle, sheep and goats) by the Rwanda Agriculture and Animal Resources Development Board (RAB) and screened for RVFV using the RealStar® RVFV RT-PCR Kit 1.0 (Altona Diagnostics). Positive blood samples were then selected for Nanopore sequencing during two capacity building workshops in Tanzania and Rwanda in October 2024 and 2025 respectively, with local follow-up sequencing onsite in Rwanda.

In October 2024, ten RVFV PCR-positive samples collected from livestock during the 2024 outbreak were randomly selected and made available by the Rubirizi Veterinary Virology Laboratory within RAB for metagenomic sequencing as described previously [9] (Supplementary Materials). Following the first metagenomic sequencing results, a pan-amplicon scheme was designed using PrimalScheme v3.2.3 (https://github.com/aresti/primalscheme), along with reference genomes (>90% segment coverage) obtained from the BV-BRC database (Table S1). The design produced 65 overlapping primers with 400 bp amplicon length in two separate pools (Table S2, Fig. S1). In October 2025, retrospective samples collected from livestock during the 2022 (*n* = 14) and 2024 outbreaks (n = 1), along with four samples collected in 2025 were selected for amplicon sequencing (Supplementary Materials). This selection was done based on sample availability. Sequencing was performed on site using the MinION device (Mk1B) and basecalling was performed using Dorado v7.9.8 (Supplementary Materials).

## 3 Results and discussion

**Fig. 1:**
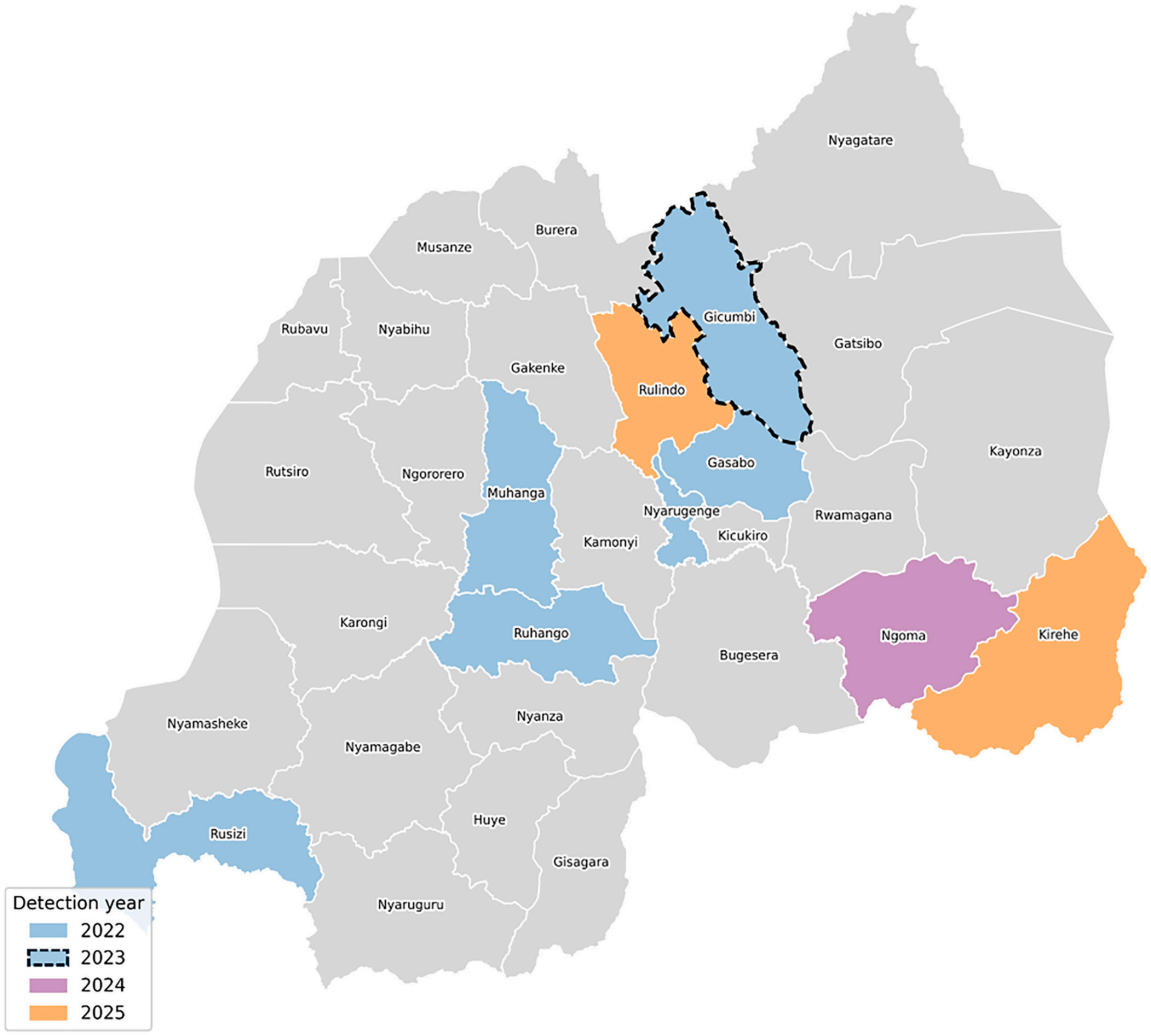
RVFV detection by district in Rwanda between 2022 and 2025. Following positive laboratory confirmation in 2024 in cattle, sheep and goats, Rwanda Agriculture and Animal Resources Development Board reported a RVF outbreak and established several immediate measures to prevent the spread of the disease to other regions [8]. These measures included mass testing and vaccination efforts. A total of 111,170 animals were vaccinated in 2024 (35,089 cattle, 73,795 goats, and 2286 sheep) increasing to 1,484,133 animals (1,051,857 cattle, 368,847 goats and 63,429 sheep) in 2025 using the thermostable Clone 13 vaccine (the total number of vaccinations per district, the total number of farms per district and total number of animals vaccinated per farm was not available) - Personal communication from the Chief Veterinary Officer, National Veterinary Reference Laboratory in Rubirizi, Rwanda, December 2025. The dashed line indicates multiple detection years with RVFV identified in Gicumbi in 2022 and 2023. A more detailed overview of RVFV cases in Rwanda between 2022 and 2025, including districts tested and the percent positivity is represented in Table S3.

RVFV surveillance in Rwanda between 2022 and 2025 indicated low overall prevalence, potentially reflecting limited testing, but had increasing positivity over time, with outbreaks affecting multiple livestock species across diverse regions (Fig. 1, Table S3). Monitoring multi-species livestock populations is critical as small ruminants can also often act as sensitive sentinel systems for human spillover.

In total, we generated nine near-complete genomes (≥95% in all three segments) from bovine samples using either metagenomic or amplicon-based sequencing, five of which were from 2022, one from 2024 and three from 2025 (Table 1). Among the partial genomes (≥80%), we generated 10 near-complete L segments, 10 near-complete M segments and 9 near-complete S segments. Overall, amplicon sequencing generated a coverage percentage of 47.33–100% (Segment S), 79.87–100% (Segment M) and 60.01–100% (Segment L) from samples with varying viral loads (Ct 15.8–30.8) (Fig. S2). We did observe one amplicon dropout in Segment M (299 bps at approx. Position 2188–2487) and one in Segment S (570 bps at approx. Position 549–1119) (Fig. S3).

**Table 1:** Overview of selected RVFV cases (2022–2025) using metagenomics and amplicon sequencing.

Sample name	Species	Collection year	Region	Ct	Sequencing approach	Coverage (%) (x6 minimum depth)		
						Seg S	Seg M	Seg L
RW_Muhanga_Bovine_2022 _1	Bovine	2022	Muhanga	24	WGS	83.47*	92.12*	100*
RW_Nyarugenge_Bovine_2022_2	Bovine	2022	Nyarugenge	26.2	WGS	74.19	91.69*	100*
RW_Nyarugenge_Bovine_2022_3	Bovine	2022	Nyarugenge	27.1	WGS	65.35	91.49*	94.6*
RW_Nyarugenge_Bovine_2022_4	Bovine	2022	Nyarugenge	23.9	WGS	88.64*	99.66*	100*
RW_Nyarugenge_Bovine_2022_5	Bovine	2022	Nyarugenge	26.1	WGS	82.09*	93.31*	98.73*
RW_Muhanga_Bovine_2022_6	Bovine	2022	Muhanga	30.8	WGS	47.33	79.87	60.01
RW_Ruhango_Bovine_2022_7	Bovine	2022	Ruhango	30	WGS	74.25	91.73*	100*
RW_Nyarugenge_Bovine_2022_8	Bovine	2022	Nyarugenge	19.3	WGS	95.72*	100*	100*
RW_Gasabo_Bovine_2022_9	Bovine	2022	Gasabo	20.9	WGS	95.66*	100*	100*
RW_Nyarugenge_Bovine_2022_10	Bovine	2022	Nyarugenge	23.4	WGS	98.83*	100*	100*
RW_Gicumbi_Bovine_2022_11	Bovine	2022	Gicumbi	26.3	WGS	83.53*	92.12*	100*
RW_Rusizi_Bovine_2022_12	Bovine	2022	Rusizi	23.1	WGS	96.8*	100*	100*
RW_Gicumbi_Bovine_2022_13	Bovine	2022	Gicumbi	16.9	WGS	97.1*	100*	100*
RW_Nyarugenge_Bovine_2022_14	Bovine	2022	Nyarugenge	22.9	WGS	82.77*	91.56*	100*
RW_Ngoma_Bovine_2024_15	Bovine	2024	Ngoma	35	Metagenomics	92.8*	66	24.6
RW_Ngoma_Bovine_2024_16	Bovine	2024	Ngoma	26.9	Metagenomics	63.5	33	29.9
RW_Ngoma_Bovine_2024_17	Bovine	2024	Ngoma	22	Metagenomics	98.1*	97.8*	96.6*
RW_Ngoma_Bovine_2024_18	Bovine	2024	Ngoma	33.6	Metagenomics	78.9	45.6	24.5
RW_Ngoma_Bovine_2024_19	Bovine	2024	Ngoma	37.2	Metagenomics	92.6*	59.2	55
RW_Ngoma_Caprine_2024_20	Caprine	2024	Ngoma	30.7	Metagenomics	42.4	22	1.2
RW_Ngoma_Caprine_2024_21	Caprine	2024	Ngoma	29.9	Metagenomics	N/A	N/A	N/A
RW_Ngoma_Ovine_2024_22	Ovine	2024	Ngoma	27.3	Metagenomics	N/A	N/A	1.36
RW_Ngoma_Ovine_2024_23	Ovine	2024	Ngoma	35.5	Metagenomics	61.3	56.2	N/A
RW_Ngoma_Ovine_2024_24	Ovine	2024	Ngoma	36	Metagenomics	N/A	N/A	N/A
RW_Ngoma_Bovine_2024_25	Bovine	2024	Ngoma	24	WGS	85.91*	92.15*	97.31*
RW_Rulindo_Bovine_2025_26	Bovine	2025	Rulindo	15.8	WGS	98.83*	100*	100*
RW_Rulindo_Bovine_2025_27	Bovine	2025	Rulindo	18.8	WGS	97.4*	100*	100*
RW_Kirehe_Bovine_2025_28	Bovine	2025	Kirehe	20.01	WGS	97.41*	99.26*	100*
RW_Kirehe_Bovine_2025_29	Bovine	2025	Kirehe	N/A	WGS	65.56	91.26*	95.28*

S; Small, M; Medium, L; Large, Seg; segment, WGS; whole-genome sequencing. *Indicates sequences used in phylogenetic analysis.

**Fig. 2:**
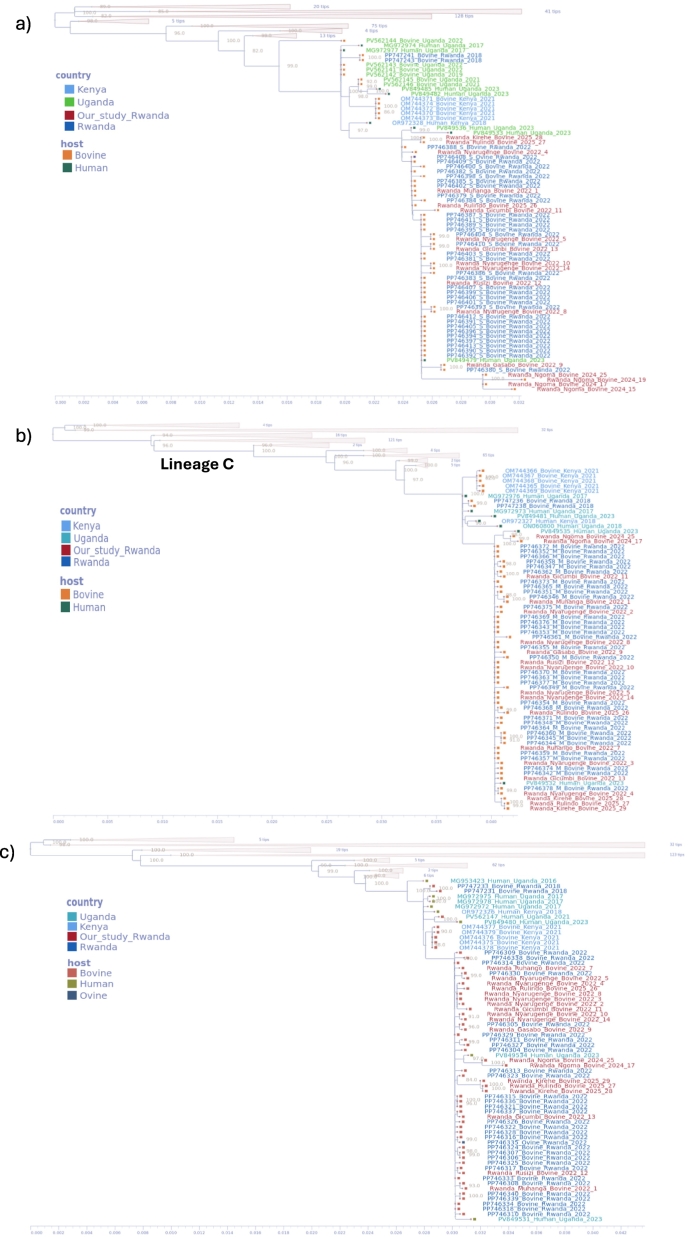
Phylogenetic analysis was performed for all RVFV segments. Reference sequences were downloaded from the BV-BRC database (https://www.bv-brc.org/), along with sequences from the 2022 outbreak in Rwanda [7]. Complete phylogenetic trees for each segment can be found in Fig. S4-S6. Fig. 2a-c illustrates a zoomed-in view of our cluster of interest for higher resolution (A) Segment S, (B) Segment M, (C) Segment L. Branch lengths are expressed as nucleotide substitutions per site and represent accumulated genetic divergence across the full phylogeny, rather than the annual substitution rate. Sequences with ≥ 80% coverage were included in the phylogeny to provide a more representative view of the genetic diversity and relationships within the dataset.

We were able to generate 55 RVFV genome segments from 21 individuals (≥80% genome coverage) for subsequent phylogenetic analysis using a rapidly designed amplicon assay using PrimalScheme for outbreak investigation. We generated three phylogenies for each RVFV segment (Figs. 2a-c, S4-S6). All our obtained sequences formed a cluster within lineage C, uniquely with sequence data from Rwanda, with a most recent common ancestor (MRCA) estimated to originate from 2021 (95% confidence intervals) (S and L segments) and 2022 (95% confidence intervals) (M segment). This suggests that 2024 and 2025 cases may have originated from a single common introduction followed by local or regional circulation. Indeed, the 2024 outbreak primarily occurred within Rwanda's Eastern Province, a region highly predisposed for transmission, with several lakes and low altitudes ideal for mosquito proliferation [10]. Meanwhile, sequences from 2025 formed more sporadic regional clusters within the 2022 outbreak, distinct from the 2024 outbreak, additionally suggesting low-level local persistence. This pattern could be compatible with sustained circulation of the original outbreak lineage, which is estimated to have emerged in mid-2021 based on the MRCA analysis. Whether maintenance of virus circulation involves undetected presence in livestock or in wild animal reservoirs remains to be determined. Periodic spillover events may have resulted in distinct epidemic clusters or transmission chains, which could explain why the 2024 and 2025 sequences cluster separately and branch from the 2022 lineage, rather than forming a shared monophyletic group. A caveat is that the interpretation of phylogenetic analysis is critically dependent on the availability of sequence data from the wider region, and for instance we cannot rule out new or repeated cross border introductions. RVFV sequences isolated within Rwanda between 2022 and 2025 also showed a close phylogenetic relationship with those isolated from Uganda in 2023 and additional sequences from neighbouring countries might reveal further relationships. Moreover our obtained sequences did not cluster with any vaccine strains within lineage E, comparatively to the 2022 outbreak, or with the 2025 outbreaks in Senegal and Mauritania within lineage H [11].

Low genomic diversity has been previously observed for RVFV with studies indicating the virus has a limited tolerance for mutations [12]. Overall, the observed substitution rate was 2.78 × 10^−4^ (0.46 subs/genome/year), 2.67 × 10^−4^ (1.02 subs/genome/year) and 2.367 × 10^−4^ (1.52 subs/genome/year) for S, M and L segments respectively. These rates are similar to previously reported rates of 5.1 × 10^−4^ (S segment), 2.7 × 10^−4^ (M segment), 1.7 × 10^−4^ (L segment) from Nsengimana and colleagues [7]. Sustained genomic surveillance and variation detection in endemic areas is essential to monitor vaccine efficiency and ensure the specificity of diagnostic real-time PCR assays. This is particularly important as vaccine campaigns and testing tend to be more reactive in response to growing outbreaks, rather than proactive nationwide preventative strategies [13]. Future studies should not only assess vaccine coverage but also evaluate post-vaccination immunity and monitor breakthrough infections.

This study had several limitations. Only a limited number of samples were available, and more representative sampling, systematic collection, sample storage, as well as vaccine status of the animals could improve our interpretation. As assay development was initiated in response to rising RVFV cases ahead of the workshop, time constraints precluded comprehensive amplicon validation and primer balancing, potentially resulting in low coverage in two genome segments. Nevertheless, our study provides a proof-of-principle approach which can be applied to other known and unknown infectious viruses within low-and-middle-income countries.

## 4 Conclusion

RVFVs circulating in 2024 and 2025 in Rwanda were closely related to viruses detected in 2022, providing evidence for ongoing circulation rather than the emergence of a novel strain. Region specific clustering could provide evidence of ongoing silent circulation within naïve livestock populations or wildlife reservoirs, underpinning the critical importance of genomic surveillance at the wildlife–livestock interface. More comprehensive genomic surveillance from East and Sub-Saharan Africa is crucial to increase outbreak resolution. Strengthening surveillance towards an integrated One Health framework, investing in research and addressing environmental triggers are crucial for future preparedness.

## CRediT authorship contribution statement

**Jean Claude Udahemuka:** Writing – review & editing, Writing – original draft, Funding acquisition, Conceptualization. **Hayley Cassidy:** Writing – review & editing, Writing – original draft, Visualization, Validation, Methodology, Investigation, Formal analysis, Data curation. **Leonard Schuele:** Writing – review & editing, Writing – original draft, Visualization, Validation, Methodology, Investigation, Formal analysis, Data curation. **Evodie Uwibambe:** Writing – review & editing, Methodology, Investigation. **Methode Gasana Ngabo:** Writing – review & editing, Data curation. **Leandre Murhula Masirika:** Writing – review & editing, Funding acquisition. **Reuben Sindayiheba:** Data curation, Methodology, Writing – review & editing. **Saria Otani:** Writing – review & editing, Funding acquisition. **Misbah Gashegu:** Writing – review & editing. **Jean-Claude Twizere:** Writing – review & editing. **Frank Aarestrup:** Writing – review & editing, Funding acquisition. **Fabrice Ndayisenga:** Writing – review & editing, Funding acquisition, Data curation. **Bas B. Oude Munnink:** Writing – review & editing, Supervision, Resources, Project administration, Funding acquisition, Conceptualization. **Marion P.G. Koopmans:** Writing – review & editing, Supervision, Resources, Project administration, Funding acquisition, Conceptualization. **Pacifique Ndishimye:** Writing – review & editing, Supervision, Resources, Project administration, Funding acquisition, Conceptualization.

## Consent for publication

Not applicable.

## Ethical statement

A research permit for this study was obtained from the Rwanda Agriculture and Animal Resources Development Board (RAB) (Ref. No. 1.11/022/022/NF/HQ). Ethical approval was granted by the Rwanda National Ethics Committee (RNEC) (Approval No. RNEC897/2025). All study procedures were conducted in accordance with the approved protocols and applicable national ethical guidelines.

## Funding

This work was funded by the Global Health EDCTP3 Joint Undertaking (Global Health EDCTP3) programme under grant agreement No. 101103059 (GREATLIFE) and 101195116 (JUA KIVU). We greatly thank the Rwanda Agriculture and Animal Resources Development Board, Kigali, Rwanda, for their collaboration during the study. Finally, we also acknowledge the EuFMD 10th FAR Funding Program for the research support they awarded to JCU, FN and PN.

## Declaration of competing interest

The authors declare the following financial interests/personal relationships which may be considered as potential competing interests: Jean Claude Udahemuka, Hayley Cassidy, Leonard Schuele, Leandre Murhula Masirika, Saria Otani, Frank Aarestrup, Bas B. Oude Munnink, Marion P.G. Koopmans and Pacifique Ndishimye report financial support was provided by EDCTP3 - JUA KIVU (101195116). Jean Claude Udahemuka, Hayley Cassidy, Leonard Schuele, Leandre Murhula Masirika, Saria Otani, Frank Aarestrup, Bas B. Oude Munnink, Marion P.G. Koopmans and Pacifique Ndishimye report financial support was provided by EDCTP3 - GREATLIFE (101103059). If there are other authors, they declare that they have no known competing financial interests or personal relationships that could have appeared to influence the work reported in this paper.

## Data Availability

All data supporting the findings of this study are available from the corresponding author upon reasonable request.

## Appendix A Supplementary data

Supplementary material Metagenomics and amplicon sequencing descriptions. Table S1. GenBank accession numbers of all reference sequences used in Nextstrain analysis. Table S2. RVFV Amplicon primers: RVFV Amplicon primers. Table S3. Overview of RVFV cases in Rwanda between 2022-2025. Figure S1. Rift Valley fever virus Primer overlapping regions. A) Segment L, B) Segment M, C) Segment SFigure S2. Ct values compared to percentage coverage. Figure S3. Example coverage patterns for amplicon sequencing. A) Segment S, B) Segment M, C) Segment L. Figure S4. Phylogenetic visualization was performed using PearTree for the RVFV S segment. Figure S5. Phylogenetic visualization was performed using PearTree for the RVFV M segment. Figure S6. Phylogenetic visualization was performed using PearTree for the RVFV L segment.
