# Supplementary material for: Persistent circulation of Rift Valley fever virus lineage C in Rwanda, 2022–2025
Source: One Health. 2026 Aug 1;23:101529. doi: 10.1016/j.onehlt.2026.101529 (PMC13471693; doi:10.1016/j.onehlt.2026.101529)
Supplement: Supplementary file 1 [file mmc1.docx]

### **Supplementary Materials**

*Metagenomics sequencing*

Total nucleic acids were extracted using the High Pure Total Nucleic Acid Kit (Roche). Extracted nucleic acids were treated with 4U of TURBO DNase (Thermo Fisher) at 37 °C for 30 minutes followed by heat inactivation following the manufacturer’s instructions. For viral metagenomics, RNA was randomly amplified using a Sequence-Independent Single-Primer (SISPA) approach (1,2). A detailed protocol is available via the GREAT LIFE protocol repository (https://www.globalsurveillance.eu/-/media/sites/global_surveillance/gl-protocols/protocol-for-viral-metagenomics-sequencing-using-ont.pdf). Briefly, first strand cDNA synthesis was performed using Super Script IV (Thermo Fisher), followed by second strand synthesis using Sequenase v2 (Thermo Fisher). The cDNA was amplified with LongAmp Polymerase (New England Biolabs).

Sequencing libraries were prepared using the Native Barcoding Kit 24 v14 (SQK-NBD114.24) (Oxford Nanopore Technologies) and sequenced on a R.10.4.1 MinION flowcell and Mk1B MinION device. Sequencing reads were basecalled using the super-high accuracy model on Dorado v7.9.8. (ONT). Sequencing reads were trimmed by fastp v0.20.1 (<https://github.com/OpenGene/fastp>) (min read length set to 200bp and Q score cutoff set to 8) and cutadapt v3.0 (<https://github.com/marcelm/cutadapt>). Minimap2 v2.28 (<https://github.com/lh3/minimap2>) was used to remove host reads, followed by taxonomic classification with KRAKEN2 [v.2.17.1](http://v.xxx) (<https://github.com/jenniferlu717/KrakenTools>) and further confirmation of invertebrate hists using NCBI BLAST. Viral consensus sequences were generated with Minimap2 (x3 coverage depth) and Samtools v1.12 (<https://github.com/samtools>).

*Amplicon sequencing*

Random priming was first performed using 50 μM random hexamers, 10 mM dNTP and 6.5 μl of extracted RNA and incubated at 65°C for 5 min followed by snap cooling at 4°C for 2 min. cDNA was then synthesized using SuperScript IV reverse transcriptase, superscript IV buffer (5x) and DTT (100mM) (Invitrogen, Carlsbad, CA, USA) and incubated at 23°C for 10 min, 50°C for 20 min and then 80°C for 10 min. Amplicons were then generated using the Q5® High-Fidelity DNA Polymerase kit, dNTP mix (10nM) and each respective primer pool (10 μM) (Integrated DNA Technologies, Coralville, IA, USA) (Table S2). PCR conditions were 98°C for 45 s, followed by 35 cycles of 98°C for 15 s and 65°C for 5 min. Sequencing libraries were generated using 100-150ng as input for the Native Barcoding Kit 24 v14 (SQK-NBD114.24) (ONT) and sequenced on a R10.4.1 flowcell (FLO-MIN114) (ONT) on a MinION Mk1B device.

Basecalling (super-high accuracy) was performed using Dorado v7.9.8. Primer sequences were trimmed by Ivar v1.4.4 (<https://github.com/andersen-lab/ivar>) followed by read trimming using fastp v0.20.1 (min read length set to 200bp and Q score cut-off set to 8). Consensus sequences were generated using Minimap2 (coverage depth set to x6, with allele frequency set to 0.05) and an inhouse reference database (Table S1). MAFFT v7.526 (<https://github.com/GSLBiotech/mafft>) was used for alignment, followed by IQ tree (v3.0.1) ( <https://github.com/iqtree/iqtree3>) for phylogenetic analysis and PearTree v1.2.2 (<https://github.com/artic-network/peartree/releases>) for visualization. Augur v28.0 (https://github.com/augurproject) on Nextstrain (<https://github.com/augurproject>) was used for evolutionary analysis. The RVFV typing tool was used for typing the glycoprotein gene (Gn) (M segment). RVFV genome sequences were deposited onto GenBank.

### Supplementary Tables and Figures

*Table S1. GenBank accession numbers of all reference sequences used in Nextstrain analysis*

| **S segment references** | **M segment references** | **L segment references** |
| --- | --- | --- |
| PP746382 | PP746342 | PP746304 |
| PP746399 | PP746354 | PP746336 |
| PP746412 | PP746369 | PP746315 |
| PP746387 | PP746364 | PP746322 |
| PP746400 | PP746376 | PP746337 |
| PP746403 | PP746374 | PP746326 |
| PP746383 | PP746365 | PP746328 |
| PP746389 | PP746353 | PP746329 |
| PP746379 | PP746352 | PP746313 |
| PP746395 | PP746366 | PP746324 |
| PP746401 | PP746355 | PP746335 |
| PP746402 | PP746378 | PP746317 |
| PP746407 | PP746368 | PP746311 |
| PP746410 | PP746351 | PP746334 |
| PP746386 | PP746371 | PP746318 |
| PP746384 | PP746373 | PP746325 |
| PP746398 | PP746345 | PP746314 |
| PP746404 | PP746347 | PP746340 |
| PP746381 | PP746372 | PP746316 |
| PP746409 | PP746348 | PP746339 |
| PP746411 | PP746358 | PP746309 |
| PP746392 | PP746360 | PP746305 |
| PP746393 | PP746343 | PP746307 |
| PP746380 | PP746362 | PP746308 |
| PP746408 | PP746344 | PP746306 |
| JQ820482 | PP746375 | PP746327 |
| EU574057 | PP746349 | PP746310 |
| EU574058 | PP746361 | PP746338 |
| EU574059 | PP746346 | PP746330 |
| EU574060 | PP746350 | PP746323 |
| EU574061 | EU574042 | MT561464 |
| EU574062 | EU574041 | MT561461 |
| EU574063 | EU574044 | OM744375 |
| EU574064 | EU574043 | OM744376 |
| EU574065 | EU574045 | OM744377 |
| EU574066 | EU574046 | OM744378 |
| EU574067 | EU574047 | OM744379 |
| EU574068 | EU574048 | JQ820483 |
| EU574069 | EU574049 | MG659820 |
| EU574070 | EU574050 | MG659819 |
| EU574071 | EU574051 | MG659821 |
| EU574072 | EU574052 | MG659822 |
| EU574073 | EU574053 | MG659823 |
| EU574074 | EU574054 | MG659824 |
| EU574075 | EU574055 | MG659825 |
| EU574076 | EU574056 | MG659826 |
| EU574077 | MT561460 | MG659827 |
| EU574078 | OM744365 | MG659828 |
| EU574079 | OM744366 | MG659829 |
| EU574080 | OM744367 | MG659831 |
| EU574081 | OM744369 | MG659830 |
| EU574082 | OM744368 | MG659832 |
| EU574083 | MG659733 | MG659834 |
| EU574084 | MG659734 | MG659833 |
| EU574085 | MG659735 | MG659835 |
| EU574086 | MG659736 | MG659836 |
| EU574087 | MG659737 | MG659837 |
| EU709747 | MG659738 | MG659838 |
| EU709748 | MG659739 | MG659839 |
| JQ820472 | MG659740 | MG659840 |
| JQ820474 | MG659741 | MG659841 |
| JQ820473 | MG659742 | MG659842 |
| JQ820475 | MG659743 | MG659843 |
| JQ820476 | MG659744 | MG659844 |
| MT561462 | MG659745 | MG659845 |
| MT561459 | MG659747 | MG659846 |
| OM744370 | MG659746 | MG659847 |
| OM744371 | MG659748 | MG659849 |
| OM744372 | MG659749 | MG659848 |
| OM744373 | MG659750 | JQ820484 |
| OM744374 | MG659752 | MG659850 |
| KU167025 | MG659751 | MG659852 |
| MG659907 | MG659753 | MG659853 |
| MG659908 | MG659754 | MG659851 |
| MG659905 | MG659756 | MG659854 |
| MG659906 | MG659755 | MG659855 |
| MG659909 | MG659757 | MG659856 |
| MG659910 | MG659758 | MG659857 |
| MG659911 | MG659759 | MG659858 |
| MG659912 | MG659760 | MG659859 |
| MG659913 | MG659761 | MG659860 |
| MG659914 | MG659762 | MG659861 |
| MG659916 | MG659763 | MG659862 |
| MG659915 | MG659764 | MG659863 |
| MG659917 | MG659765 | MG659864 |
| MG659918 | MG659766 | MG659865 |
| MG659919 | MG659767 | MG659866 |
| MG659920 | MG659768 | MG659868 |
| MG659921 | MG659770 | MG659867 |
| MG659922 | MG659771 | MG659869 |
| MG659923 | MG659769 | MG659870 |
| MG659924 | MG659772 | MG659871 |
| MG659925 | MG659773 | MG659872 |
| MG659926 | MG659774 | MG659873 |
| MG659927 | MG659775 | MG659874 |
| MG659928 | MG659777 | MG659875 |
| MG659929 | MG659776 | MG659876 |
| MG659930 | MG659778 | MG659877 |
| MG659931 | MG659779 | MG659878 |
| MG659932 | MG659780 | MG659879 |
| MG659933 | MG659782 | MG659880 |
| MG659934 | MG659781 | MG659882 |
| MG659935 | MG659783 | MG659881 |
| MG659936 | MG659784 | MG659883 |
| MG659937 | MG659785 | MG659884 |
| MG659938 | MG659786 | MG659885 |
| MG659939 | MG659787 | MG659886 |
| MG659940 | MG659788 | MG659887 |
| MG659941 | MG659789 | MG659888 |
| MG659942 | MG659790 | MG659889 |
| MG659943 | MG659792 | MG659890 |
| MG659944 | MG659791 | MG659891 |
| MG659946 | MG659793 | MG659892 |
| MG659945 | MG659794 | MG659893 |
| MG659947 | MG659795 | MG659894 |
| MG659948 | MG659796 | MG659895 |
| MG659949 | MG659798 | MG659896 |
| MG659950 | MG659799 | MG659897 |
| MG659951 | MG659800 | MG659898 |
| MG659952 | MG659801 | MG659899 |
| MG659953 | MG659802 | MG659900 |
| MG659954 | MG659803 | MG659901 |
| MG659955 | MG659797 | MG659902 |
| MG659956 | MG659804 | MG659904 |
| MG659957 | MG659805 | MG659903 |
| MG659958 | MG659806 | KU167027 |
| MG659959 | MG659807 | JQ820485 |
| MG659960 | MG659810 | MZ513011 |
| MG659961 | MG659808 | MZ513012 |
| MG659962 | MG659809 | MZ513013 |
| MG659963 | MG659811 | KY126665 |
| MG659965 | MG659812 | KY126666 |
| MG659964 | MG659813 | KY126668 |
| MG659966 | MG659814 | KY126667 |
| MG659967 | MG659815 | KY126669 |
| MG659968 | MG659816 | KY126671 |
| MG659969 | MG659817 | KY126670 |
| MG659971 | MG659818 | KY126672 |
| MG659970 | KU167026 | KY126673 |
| MG659972 | KY126690 | KY126674 |
| MG659973 | KY126691 | KY126675 |
| MG659974 | KY126692 | KY126677 |
| MG659975 | KY126693 | KY126676 |
| MG659976 | KY126694 | KY126678 |
| MG659977 | KY126696 | KY126679 |
| MG659978 | KY126695 | KY126680 |
| MG659979 | KY126698 | KY126681 |
| MG659980 | KY126697 | KY126683 |
| MG659981 | KY126700 | KY126682 |
| MG659982 | KY126699 | KY126684 |
| MG659983 | KY126701 | KY126685 |
| MG659984 | KY126702 | KY126686 |
| MG659986 | KY126703 | KY126687 |
| MG659985 | KY126704 | KY126688 |
| MG659987 | KY126705 | KY126689 |
| MG659988 | KY126706 | JQ820486 |
| MG659990 | KY126707 | OM744403 |
| MG659989 | KY126708 | OP146107 |
| KY196487 | KY126709 | OP146110 |
| KY196488 | KY126710 | OR972326 |
| KY196489 | KY126711 | MG953422 |
| KY196490 | KY126712 | MG953423 |
| KY196492 | KY126713 | MG972972 |
| KY196491 | KY126714 | MG972975 |
| KY196493 | OM744404 | MG972978 |
| KY196494 | ON060800 | KU978779 |
| KY196495 | JQ820487 | KU978780 |
| KY196496 | OP146105 | KU978781 |
| KY196497 | OP146108 | KX096938 |
| KY196498 | OP146111 | KX096941 |
| KY196499 | OR125122 | KX609031 |
| KY196500 | MG953419 | KX611605 |
| KY196501 | OR972327 | KX632066 |
| KY196502 | JQ820488 | KJ782454 |
| KY196503 | JQ820489 | KJ782457 |
| KY196504 | MG972973 | KX944849 |
| KY196505 | JQ820490 | KX944850 |
| KY196506 | MG972976 | KX944851 |
| KY196508 | JQ820491 | KX944852 |
| KY196509 | KU978776 | KX944853 |
| KY196510 | KU978778 | KX944854 |
| KY196511 | KX096939 | KX944855 |
| KY196507 | KX096942 | KX944856 |
| OM744405 | KX609032 | KX944857 |
| OP146106 | KX611606 | KX944858 |
| OP146109 | KX632067 | KX944859 |
| OP146112 | KJ782453 | KX944860 |
| OR972328 | KJ782456 | KX944861 |
| OR528950 | KX944826 | KX944862 |
| OR528951 | KX944827 | KX944863 |
| OR528953 | KX944828 | KX944864 |
| OR528952 | KX944829 | KX944865 |
| MG953425 | KX944830 | KX944866 |
| MG953426 | KX944831 | KX944868 |
| OR805807 | KX944833 | KX944869 |
| MG972974 | KM210509 | MG649050 |
| MG972977 | KX944834 | KX944870 |
| JQ840745 | KX944835 | KX944871 |
| JQ840746 | KX944836 | HM586953 |
| KU978773 | KX944837 | HM586954 |
| KX096940 | KX944838 | HM586955 |
| KX096943 | KX944839 | HM586956 |
| KX611607 | KX944840 | HM586957 |
| KJ782452 | KX944841 | HM586958 |
| KJ782455 | KX944842 | HM586959 |
| KX944803 | KX944843 | HM586960 |
| KX944804 | KX944844 | MG649051 |
| KX944805 | KX944845 | HM586961 |
| KX944806 | KX944846 | HM586962 |
| KX944807 | KX944847 | HM586963 |
| KX944808 | KX944848 | MG649052 |
| KX944809 | HM586964 | MG649053 |
| KX944810 | HM586965 | DQ375395 |
| KX944811 | HM586966 | DQ375396 |
| KX944812 | HM586967 | DQ375397 |
| KX944813 | HM586968 | DQ375398 |
| KX944816 | HM586969 | DQ375399 |
| KX944817 | HM586970 | DQ375400 |
| KX944818 | HM586971 | DQ375401 |
| KX944819 | HM586972 | DQ375402 |
| KX944820 | HM586973 | DQ375403 |
| KX944821 | HM586974 | DQ375404 |
| KX944822 | MG649054 | DQ375405 |
| KX944823 | MG649055 | DQ375406 |
| KX944825 | MG649056 | DQ375407 |
| KM210508 | MG649057 | DQ375408 |
| JQ820477 | NC_014396 | DQ375409 |
| HM586975 | HQ009512 | DQ375410 |
| HM586976 | DQ380183 | DQ375411 |
| HM586977 | DQ380184 | DQ375412 |
| HM586978 | DQ380185 | DQ375413 |
| HM586979 | DQ380186 | DQ375414 |
| HM586980 | DQ380187 | DQ375415 |
| HM586981 | DQ380188 | DQ375416 |
| HM586982 | DQ380189 | DQ375417 |
| HM586983 | DQ380191 | DQ375418 |
| HM586984 | DQ380194 | NC_014397 |
| JQ820478 | DQ380195 | DQ375419 |
| NC_014395 | DQ380196 | DQ375420 |
| DQ380143 | DQ380197 | DQ375421 |
| DQ380144 | DQ380198 | DQ375422 |
| DQ380145 | DQ380199 | DQ375423 |
| DQ380146 | DQ380200 | DQ375424 |
| DQ380149 | DQ380201 | DQ375426 |
| DQ380151 | DQ380202 | DQ375425 |
| DQ380152 | DQ380203 | DQ375428 |
| DQ380153 | DQ380204 | DQ375429 |
| DQ380154 | DQ380205 | DQ375430 |
| DQ380155 | DQ380206 | DQ375431 |
| DQ380156 | DQ380207 | DQ375432 |
| DQ380157 | DQ380209 | DQ375433 |
| DQ380158 | DQ380210 | DQ375434 |
| DQ380159 | DQ380211 | JF311368 |
| DQ380160 | DQ380212 | JF311369 |
| DQ380161 | DQ380214 | JF311370 |
| DQ380162 | DQ380215 | JF311371 |
| DQ380163 | DQ380216 | JF311372 |
| DQ380164 | DQ380217 | JF311373 |
| DQ380165 | DQ380218 | JF311374 |
| DQ380166 | DQ380219 | JF311375 |
| DQ380167 | DQ380220 | JF311376 |
| DQ380168 | DQ380221 | JF326186 |
| DQ380169 | DQ380222 | JF326187 |
| DQ380170 | EF460404 | JF326188 |
| DQ380171 | EF467177 | JF326189 |
| DQ380172 | EF467178 | JF326190 |
| DQ380173 | JF309200 | JF784386 |
| DQ380174 | JF311377 | JQ068144 |
| DQ380175 | JF311378 | EU574004 |
| DQ380177 | JF311379 | EU574005 |
| DQ380178 | JF311380 | EU574006 |
| DQ380179 | JF311381 | EU574007 |
| DQ380180 | JF311382 | EU574008 |
| DQ380181 | JF311383 | EU574009 |
| JQ820479 | JF311384 | EU574010 |
| JF311386 | JF311385 | EU574011 |
| JF311387 | JF326191 | EU574012 |
| JF311388 | JF326192 | EU574013 |
| JF311389 | JF326193 | EU574014 |
| JF311390 | JF326194 | EU574015 |
| JF311391 | JF326195 | EU574016 |
| JF311392 | JF784387 | EU574017 |
| JF311393 | JQ068143 | EU574018 |
| JF311394 | EU574031 | EU574019 |
| JQ820480 | EU574032 | EU574020 |
| JF326196 | EU574033 | EU574021 |
| JF326197 | EU574034 | EU574022 |
| JF326198 | EU574035 | EU574023 |
| JF326199 | EU574036 | EU574024 |
| JF326200 | EU574037 | EU574025 |
| JF326201 | EU574038 | EU574026 |
| JF326202 | EU574039 | EU574027 |
| JF326203 | PP747236 | EU574028 |
| JF326204 | PP747238 | EU574029 |
| JF784388 | DQ380213 | EU574030 |
| EU312103 | DQ380193 | PP747231 |
| EU312104 | PP746359 | PP747233 |
| EU312105 | PP746363 | OP146104 |
| EU312106 | PP746357 | PP746321 |
| EU312107 | PP746367 | PP746331 |
| EU312108 | PP746370 | PP746332 |
| EU312109 | PP746377 | PP746333 |
| EU312110 | PP746356 | PP746312 |
| EU312111 | PV849481 | PP746319 |
| EU312112 | PV849532 | PP746320 |
| EU312113 | PV849535 | PV849534 |
| EU312114 | PX580509 | PV849531 |
| EU312115 |  | PV849480 |
| EU312116 |  | PV562147 |
| EU312117 |  | PX580510 |
| EU312118 |  |  |
| EU312119 |  |  |
| EU312120 |  |  |
| EU312121 |  |  |
| EU312122 |  |  |
| EU312123 |  |  |
| EU312124 |  |  |
| EU312125 |  |  |
| EU312126 |  |  |
| EU312127 |  |  |
| EU312128 |  |  |
| EU312130 |  |  |
| EU312131 |  |  |
| EU312132 |  |  |
| EU312133 |  |  |
| EU312134 |  |  |
| EU312135 |  |  |
| EU312136 |  |  |
| EU312137 |  |  |
| EU312138 |  |  |
| EU312139 |  |  |
| EU312140 |  |  |
| EU312141 |  |  |
| EU312142 |  |  |
| EU312143 |  |  |
| EU312144 |  |  |
| EU312145 |  |  |
| EU312146 |  |  |
| EU312147 |  |  |
| JQ820481 |  |  |
| PP747241 |  |  |
| PP747243 |  |  |
| DQ380182 |  |  |
| PP746388 |  |  |
| PP746397 |  |  |
| PP746406 |  |  |
| PP746390 |  |  |
| PP746394 |  |  |
| PP746396 |  |  |
| PP746405 |  |  |
| PP746413 |  |  |
| PP746385 |  |  |
| PP746391 |  |  |
| PV849536 |  |  |
| PV849533 |  |  |
| PV849485 |  |  |
| PV849482 |  |  |
| PV849479 |  |  |
| PV562143 |  |  |
| PV562141 |  |  |
| PV562142 |  |  |
| PV562145 |  |  |
| PV562146 |  |  |
| PV562144 |  |  |

*Table S2. RVFV Amplicon primers*

| **Name** | **Position** | **Position** | **Pool** | **Sense** | **Sequence** |
| --- | --- | --- | --- | --- | --- |
| SegL_RVFV_1_A | 22 | 43 | 1 | + | ACACAAAGGCGCCCAATCATG |
| SegL_RVFV_2_A | 567 | 590 | 1 | - | TGCTTCAGAGTCCTCTAGCTCCA |
| SegL_RVFV_3_B | 459 | 487 | 2 | + | CCATGACTAAACTCGCTAAGTATGAGGT |
| SegL_RVFV_4_B | 1009 | 1031 | 2 | - | CCAGGGAGGCAGCTGAATAGTT |
| SegL_RVFV_5_A | 890 | 920 | 1 | + | GGGAATGACAAAGTTCTGAGATTTTCAAAA |
| SegL_RVFV_6_A | 1440 | 1471 | 1 | - | TCTTTATCACAAGAGAGAGGGTTGTATAGAT |
| SegL_RVFV_7_B | 1333 | 1360 | 2 | + | GCAAATGAGAAATAGGAGTAAGCAGCC |
| SegL_RVFV_8_B | 1883 | 1906 | 2 | - | TCTGCCCAGAATGAGAAGGAGGA |
| SegL_RVFV_9_A | 1777 | 1802 | 1 | + | GGTCTTCAAGCCCTACATAGATGCT |
| SegL_RVFV_10_A | 2327 | 2350 | 1 | - | GGCTCGGTCTCCTCTTCTTTGTT |
| SegL_RVFV_11_B | 2210 | 2243 | 2 | + | ATTCTTAAGAAAAAGGATGGATCTATATCCTGG |
| SegL_RVFV_12_B | 2760 | 2786 | 2 | - | ACAAATATGCATGCATCCTTGTGACT |
| SegL_RVFV_13_A | 2651 | 2678 | 1 | + | GCAGACAAGAACTACACAAGGGATAAA |
| SegL_RVFV_14_A | 3201 | 3231 | 1 | - | CATGATAAGCTTTGAAGAGATCCATCACAA |
| SegL_RVFV_15_B | 3094 | 3119 | 2 | + | GCTGATCATTAGGGGATGCTCAATG |
| SegL_RVFV_16_B | 3644 | 3667 | 2 | - | GCTGCAATCCATCTGATCGTTGG |
| SegL_RVFV_17_A | 3540 | 3564 | 1 | + | TGGGAGTGTACCTTGCCATTTACC |
| SegL_RVFV_18_A | 4090 | 4118 | 1 | - | CCTAGATCCCCACTTTAGAGAAGAGCTA |
| SegL_RVFV_19_B | 3925 | 3957 | 2 | + | AGGTTTCAGATTTAATCTCTTCAAAGCTATCA |
| SegL_RVFV_20_B | 4475 | 4505 | 2 | - | ATCCAGTGATTCTAATTCCTCAATATTCGG |
| SegL_RVFV_21_A | 4362 | 4391 | 1 | + | CTGAGTTCAACTTCTTGGAGGATTCTAAG |
| SegL_RVFV_22_A | 4912 | 4934 | 1 | - | AGCAAGGTCTTCTACACCTCGG |
| SegL_RVFV_23_B | 4806 | 4831 | 2 | + | GCAAGCCCAGAACAGTCAGAATAAC |
| SegL_RVFV_24_B | 5356 | 5380 | 2 | - | GCCCACTGCCTTATTGGAACACTC |
| SegL_RVFV_25_A | 5249 | 5272 | 1 | + | GGCTTCATGGACGGATATCAGGT |
| SegL_RVFV_26_A | 5799 | 5830 | 1 | - | AGTCTGGTTCTGTTAATTCCTTTTATCTGTC |
| SegL_RVFV_27_B | 5692 | 5717 | 2 | + | TCAAGCCCTGCAATTATTTGAGAGG |
| SegL_RVFV_28_B | 6242 | 6267 | 2 | - | TCCTGATGCTTTCTTCTGGTCTCTG |
| SegL_RVFV_29_A | 5847 | 5876 | 1 | + | TTTGTTCTGAATCTTCCCTGAGATCAAAA |
| SegL_RVFV_30_A | 6397 | 6423 | 1 | - | GACCGTCCAATATTGTAGCACTATGC |
| SegM_RVFV_1_A | 0 | 28 | 1 | + | ACACAAAGACGGTGCATTAAATGTATGT |
| SegM_RVFV_2_A | 525 | 547 | 1 | - | TCCTCCTGAGTCATCCCGTCAA |
| SegM_RVFV_3_B | 383 | 410 | 2 | + | GATGCTAAGCAAATAGGGAGAGAAACC |
| SegM_RVFV_4_B | 932 | 959 | 2 | - | TTTTGATCCATCATCCTCACTTGACTG |
| SegM_RVFV_5_A | 812 | 835 | 1 | + | AAAATGGCTTCAGTCAAGTGCCC |
| SegM_RVFV_6_A | 1362 | 1384 | 1 | - | AAAGGCTTCTTCCAGACCCCTG |
| SegM_RVFV_7_A | 1362 | 1388 | 1 | - | ACATAAAGGTTTCTTCCAGACCCCTG |
| SegM_RVFV_8_B | 1198 | 1226 | 2 | + | GCTCAAAAGCTTTGATATCTCTCAGTGC |
| SegM_RVFV_9_B | 1736 | 1762 | 2 | - | GTGTGACACTGGTAATTTATCAGGCC |
| SegM_RVFV_10_A | 1568 | 1599 | 1 | + | AGTCCTTCTACTGAGATTACACTCAAGTATC |
| SegM_RVFV_11_A | 1570 | 1599 | 1 | + | TCCTTCTACCGAGATTACACTCAAGTATC |
| SegM_RVFV_12_A | 2113 | 2136 | 1 | - | TGGAGCAAGTGGTGATTCTGGAG |
| SegM_RVFV_13_B | 1973 | 1995 | 2 | + | TGGATGGAAGGAGGTCAGTTGG |
| SegM_RVFV_14_B | 2513 | 2538 | 2 | - | GCAGATACGTGTGCACAAATAAGCA |
| SegM_RVFV_15_A | 2406 | 2433 | 1 | + | CTTCAGCAGAGTTTTCATTTGTTGGAG |
| SegM_RVFV_16_A | 2408 | 2433 | 1 | + | TCAGCAGAGTTTTCATTTGTTGGGG |
| SegM_RVFV_17_A | 2956 | 2980 | 1 | - | TCATTCCTTGTCTGTGGCAGAGAA |
| SegM_RVFV_18_B | 2839 | 2862 | 2 | + | CCTGAGTGCTCATGAATCATGCC |
| SegM_RVFV_19_B | 3358 | 3388 | 2 | - | CTATCATCAAATGGGTCAATAGCTATCAGG |
| SegM_RVFV_20_B | 3358 | 3390 | 2 | - | GCCTATCATCAAATGGATCAATAGCTATCAGA |
| SegM_RVFV_21_A | 3207 | 3230 | 1 | + | ACAATAAGGACGGGTCTCTGCAT |
| SegM_RVFV_22_A | 3205 | 3230 | 1 | + | CCACAATAAGGATGGGTCTCTGCAT |
| SegM_RVFV_23_A | 3751 | 3774 | 1 | - | CCCCACCACCCCAAATAACAACT |
| SegM_RVFV_24_A | 3751 | 3774 | 1 | - | CCCCACCACCCCAAATTACAACT |
| SegM_RVFV_25_B | 3400 | 3424 | 2 | + | GGGGGAATCAACAGTTGTGAATCC |
| SegM_RVFV_26_B | 3398 | 3424 | 2 | + | GGGGGAGAATCAATAGTTGTGAATCC |
| SegM_RVFV_27_B | 3863 | 3885 | 2 | - | ACACAAAGACCGGTGCAACTTC |
| SegS_RVFV_1_A | 0 | 24 | 1 | + | ACACAAAGACCCCCTAGTGCTTAT |
| SegS_RVFV_2_A | 524 | 548 | 1 | - | GGATAGCCTCGGTCACTATCATCC |
| SegS_RVFV_3_B | 378 | 400 | 2 | + | TGGAGAACCCTCACTGGCTTTC |
| SegS_RVFV_4_B | 928 | 950 | 2 | - | GCTGCTCAGGCCTACAAAACAG |
| SegS_RVFV_5_A | 825 | 848 | 1 | + | TGATTAGAGGTTAAGGCTGCCCC |
| SegS_RVFV_6_A | 1375 | 1403 | 1 | - | CTCATCAACAAGTACAAGCTAAAGGAGG |
| SegS_RVFV_7_B | 1125 | 1147 | 2 | + | CGGGAGAACTGCAGCAGATACA |
| SegS_RVFV_8_B | 1635 | 1664 | 2 | - | ACAATAATGGACAACTATCAAGAGCTTGC |

***Table footnotes:*** *SegL; Segment L, SegM; Segment M, SegS; Segment S, RVFV; Rift Valley fever virus*

*Table S3. Overview of RVFV cases in Rwanda between 2022-2025*

| **Districts tested** | **Collection period** | **Positive districts** | **Total no. of animal samples tested** | **Total no. of positives** | **Species** | **Case fatality** | **Percent positivity** | **Refs** |
| --- | --- | --- | --- | --- | --- | --- | --- | --- |
| All (30 districts) | March - December 2022 | Muhanga, Nyarugenge,Ruhango, Gasabo, Gicumbi, Rusizi | 1,880,591 | 1342 | Cattle (95.8%), goats (2.5%) and sheep (1.7%) | 38% | 0.07% | 3, 4 |
| Gicumbi | October 2023 | Gicumbi | 1288 | 5 | N/A | N/A | 0.38% | * |
| Gasabo, Kamonyi, Nyarugenge, Musanze, Gatsibo, Kirehe and Ngoma* | March – October  2024 | Ngoma | 2692 | 22 | Cattle (45.8%), goats (33.3%) and sheep (20.8%) | 8.30% | 0.90% | 5 |
| Muhanga, Rulindo and Kirehe | March - April and October 2025 | Rulindo and Kirehe | 936 | 18 | N/A | N/A | 1.92% | * |

***Table footnotes:*** *N/A; not available. * Personal communication from the Chief Veterinary Officer, National Veterinary Reference Laboratory in Rubirizi, Rwanda, December 2025*

A)

B)

C)

*Figure S1. Rift Valley fever virus Primer overlapping regions. A) Segment L, B) Segment M, C) Segment S*

*Figure S2. Ct values compared to percentage coverage*

A)

B)

C)

*Figure S3. Example coverage patterns for amplicon sequencing. A) Segment S, B) Segment M, C) Segment L. Two amplicon dropouts can be observed within Segment M (299bps at approx. position 2188-2487) and in Segment S (570 bps at approx. position 549-1119).*

*Figure S4. Phylogenetic visualization was performed using PearTree for the RVFV S segment. Reference sequences were downloaded from the BV-BRC database (https://www.bv-brc.org/), along with sequences from the 2022 outbreak in Rwanda (6). The rectangular dotted red box represents the cluster of interest including our consensus sequences. An enlarged view of our highlighted cluster is shown in the main article to provide higher resolution of divergence.*

*Figure S5. Phylogenetic visualization was performed using PearTree for the RVFV M segment. Reference sequences were downloaded from the BV-BRC database (https://www.bv-brc.org/), along with sequences from the 2022 outbreak in Rwanda (6). The rectangular dotted red box represents the cluster of interest including our consensus sequences. An enlarged view of our highlighted cluster is shown in the main article to provide higher resolution of divergence. We have additionally provided RVFV genotypes based on the glycoprotein gene within the M segment.*

*Figure S6. Phylogenetic visualization was performed using PearTree for the RVFV L segment. Reference sequences were downloaded from the BV-BRC database (https://www.bv-brc.org/), along with sequences from the 2022 outbreak in Rwanda (6). The rectangular dotted red box represents the cluster of interest including our consensus sequences. An enlarged view of our highlighted cluster is shown in the main article to provide higher resolution of divergence.*

*References:*

1. Greninger, Alexander L., et al. "Rapid metagenomic identification of viral pathogens in clinical samples by real-time nanopore sequencing analysis." *Genome medicine* 7.1 (2015): 99.
2. Schuele, Leonard, et al. "Metagenomic sequencing of mpox virus clade Ib lesions identifies possible bacterial and viral co-infections in hospitalized patients in eastern DRC." Microbiology spectrum 13.7 (2025): e00512-25.
3. Remera E, Rwagasore E, Nsekuye O, Semakula M, Gashegu M, Rutayisire R, Ishema L, Musanabaganwa C, Butera Y, Nsanzimana S, Muvunyi CM, Ahmed A. Rift Valley Fever Epizootic, Rwanda, 2022. Emerg Infect Dis. 2024 Oct;30(10):2191-2193. doi: 10.3201/eid3010.240264. PMID: 39320366; PMCID: PMC11431898.
4. Ishema L, Colombe S, Ndayisenga F, Uwibambe E, Van Damme E, Meudec M, Rwagasore E, Mugwaneza D, Van Bortel W, Shyaka A. One Health investigation and response to a nationwide outbreak of Rift Valley fever in Rwanda - March to December 2022. One Health. 2024 Jul 4;19:100854. doi: 10.1016/j.onehlt.2024.100854. PMID: 40206839; PMCID: PMC11980628.
5. Ministry of Agriculture and Animal Resources (Rwanda). Rwanda tackles RVF using ‘One Health’ approach. MINAGRI. Published 26 September 2024. Available at: https://www.minagri.gov.rw/updates/news-details/rwanda-tackles-rvf-using-one-health-approach (accessed 20/01/2026)
6. Nsengimana I, Juma J, Roesel K, Gasana MN, Ndayisenga F, Muvunyi CM, Hakizimana E, Hakizimana JN, Eastwood G, Chengula AA, Bett B, Kasanga CJ, Oyola SO. Genomic 196 Epidemiology of Rift Valley Fever Virus Involved in the 2018 and 2022 Outbreaks in 197 Livestock in Rwanda. Viruses. 2024 Jul 17;16(7):1148. doi: 10.3390/v16071148. PMID: 39066310; PMCID: PMC11281637.
